# X-ray tensor tomography for small-grained polycrystals with strong texture

**DOI:** 10.1107/S1600576724004588

**Published:** 2024-06-27

**Authors:** Mads Carlsen, Christian Appel, William Hearn, Martina Olsson, Andreas Menzel, Marianne Liebi

**Affiliations:** a Photon Science Division Paul Scherrer Institut 5232Villigen PSI Switzerland; b Department of Physics, Chalmers University of Technology, SE-412 96Gothenburg, Sweden; c Institute of Materials Ecole Polytechnique Fédérale de Lausanne (EPFL) 1015Lausanne Switzerland; Ecole National Supérieure des Mines, Saint-Etienne, France

**Keywords:** tensor tomography, texture analysis, wide- and small-angle X-ray scattering, SAXS, WAXS

## Abstract

Small-angle X-ray tensor tomography and the related wide-angle X-ray tensor tomography are X-ray imaging techniques that tomographically reconstruct the anisotropic scattering density of extended samples. In previous studies, these methods have been used to image samples where the scattering density depends slowly on the direction of scattering, typically modeling the directionality, *i.e.* the texture, with a spherical harmonics expansion up until order ℓ = 8 or lower. This study investigates the performance of several established algorithms from small-angle X-ray tensor tomography on samples with a faster variation as a function of scattering direction and compares their expected and achieved performance. The various algorithms are tested using wide-angle scattering data from an as-drawn steel wire with known texture to establish the viability of the tensor tomography approach for such samples and to compare the performance of existing algorithms.

## Abstract 2

The article reports an investigation and comparison of different algorithms from small-angle X-ray scattering tensor tomography for reconstructing the anisotropic scattering density of samples with fast directional variation. The different methods are tested using wide-angle scattering data from an as-drawn steel wire.

## 1. Introduction

In recent years, a method of tomographically reconstructing the scattering density of non-isotropically scattering samples has gained traction in small-angle X-ray scattering communities. This method, known as small-angle X-ray scattering tensor tomography (SASTT), has been successfully applied to study a wide range of phenomena in mineralized organic tissue, ranging from bioinspired synthetic materials (Rajasekharan *et al.*, 2018 ▸) to axon orientations in brains (Georgiadis *et al.*, 2021 ▸) and healing around bone implants (Liebi *et al.*, 2021 ▸). This method has been extended to wide-angle X-ray scattering (WAXS) from crystalline systems including hydroxy­apatite in bone (Grünewald *et al.*, 2020 ▸, 2023 ▸) and clay minerals in Pierre shale (Mürer *et al.*, 2021 ▸). Yet studies so far have been limited to materials that display weak axially symmetric texture similar to that commonly observed in SAXS.

Tensor tomography (TT) differs from other computed tomography (CT) reconstructions (Stock *et al.*, 2008 ▸; Bleuet *et al.*, 2008 ▸) by explicitly modeling and reconstructing the scattering anisotropy. The function that describes the intensity of scattering, as a function of both scattering angle and direction of scattering, is called a three-dimensional reciprocal space map (3D-RSM). In WAXS, the 3D-RSM evaluated at a fixed scattering angle, corresponding to a given Bragg reflection *hk*ℓ, is proportional to a function used in texture analysis called a pole figure. We refer to this function as 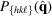, where 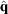 is the normalized scattering vector which we use as a variable to describe the direction of scattering. The properly normalized *P*_{*hk*ℓ}_ can be understood as the probability density of finding a reciprocal lattice vector of the {*hk*ℓ} planes parallel to the direction 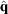. Because the pole figure is defined as a volume average over many grains, a spatially resolved reconstruction of the pole figure is only appropriate if the spatial resolution is much larger than the average grain size of the sample, or when grains are strongly deformed and have inherent mosaicity.

As-deformed metals often contain small and highly mosaic grains and strong crystallographic texture caused by processing stresses. The sample investigated here displays grain sizes on the order of hundreds of nanometres and azimuthal variation of the powder rings with a full width at half-maximum of around 10°. Owing to the deformed grains, such samples are difficult to study with non-destructive grain-mapping methods such as three-dimensional X-ray diffraction (3D-XRD) (Poulsen, 2004 ▸) and diffraction contrast tomography (Ludwig *et al.*, 2008 ▸; Johnson *et al.*, 2008 ▸), which rely on identifying individual diffraction spots in the detector images and are therefore restricted to samples containing large grains. This restriction can partially be alleviated by using a focused X-ray beam and raster-scanning the sample, as done in scanning 3D-XRD (Hayashi *et al.*, 2015 ▸). Differential-aperture X-ray microscopy (Yang *et al.*, 2004 ▸; Larson & Levine, 2013 ▸) also images grain structures by inserting and scanning an additional optical component downstream of the sample rather than using tomographic methods.

In recent years, efforts have been made to extend 3D-XRD to be able to characterize materials with a higher degree of deformation and smaller grain sizes (Henningsson *et al.*, 2020 ▸; Kutsal *et al.*, 2022 ▸). But since these methods all apply peak finding as the first analysis step, they can only work when isolated peaks stemming from individual grains can be observed on the detector.

Wide-angle X-ray scattering tensor tomography (WASTT) gets around this limitation by attempting to reconstruct not individual grains but rather the pole figures, which contain information about averages over many grains. Therefore, establishing WASTT as a viable method for such systems would make it possible to non-destructively image the microstructure of such small-grained samples, which is not possible with existing methods.

Initial studies using WASTT have focused on the 002 peak in bone apatite, which has a slow dependence on the scattering direction and strong scattering in a single direction, similar to that typically observed in SAXS (Grünewald *et al.*, 2020 ▸, 2023 ▸). This has in part to do with the extremely small crystalline domains found in bone hydroxyapatite, on the order of tens of nanometres, as well as the low multiplicity of the hexagonal 002 reflection. The samples of interest for this study differ from what has been observed in studies on bone in that the texture is stronger, *i.e.* the variation of scattering intensity with respect to the scattering angle is much faster. Therefore, a higher angular resolution is needed to properly model the pole figure.

In this paper, we apply a representative set of algorithms from SASTT on samples with fast directional variation of the reciprocal space map. We compare their performance on the basis of the number of degrees of freedom at a given angular resolution. To test the algorithms, we apply them on WAXS data from an as-drawn steel wire with known texture. The investigated sample shows narrow features in the measured diffraction patterns with an FWHM of approximately 10° in the azimuthal angle and is chosen because of its local wire-symmetric texture, which can be correlated with its geometric shape to provide an easy test case and establish the correctness of the reconstructed texture.

## 2. Experimental details

### 2.1. Experiment

**Figure 1:**
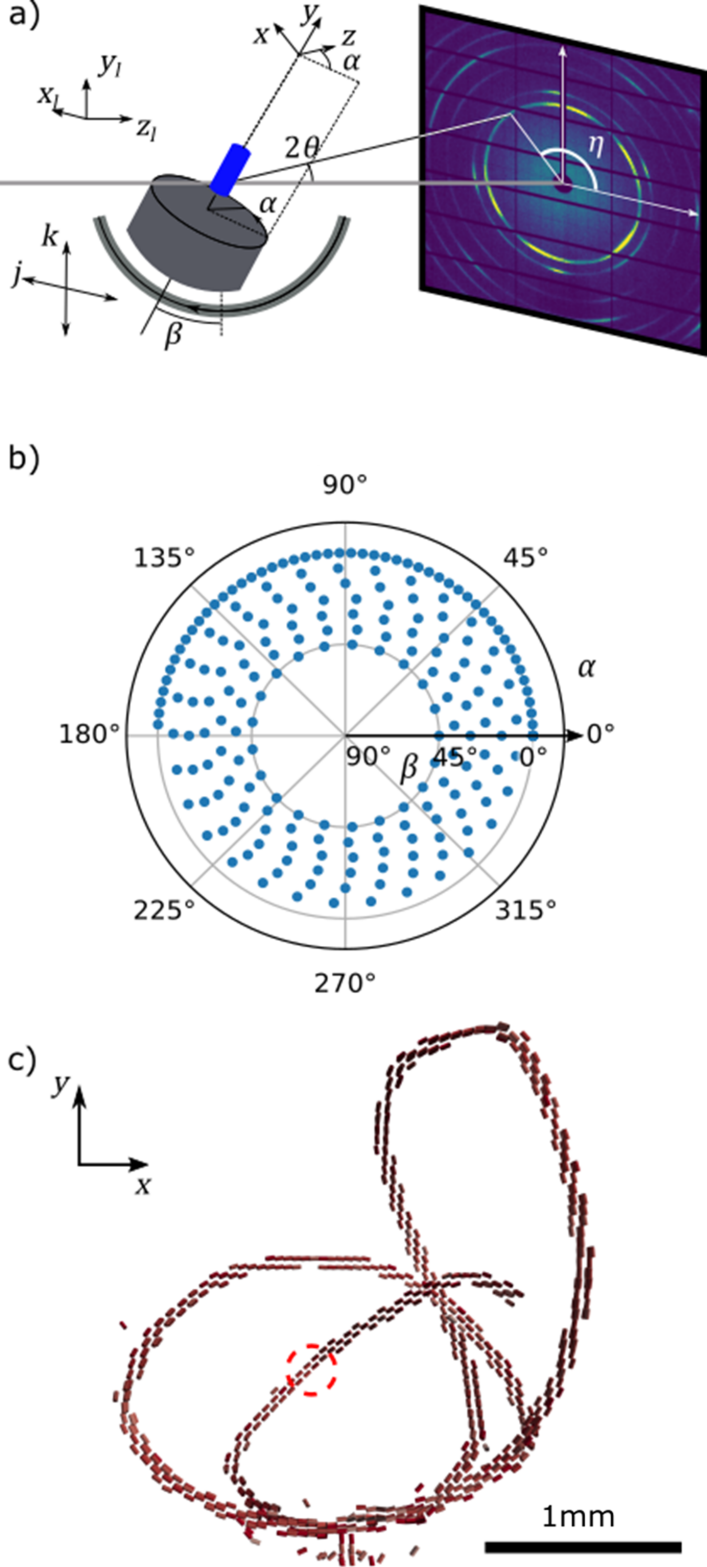
(*a*) Sketch of the experimental geometry with coordinate systems and goniometer angles indicated. The α rotation is around the laboratory *y* axis and the β rotation around the laboratory *x* axis. At α = β = 0 the laboratory and sample coordinate systems coincide. (*b*) Measured sample orientations plotted as (α, β) points. The measured directions are limited to a range of β ≤ 45° by the geometry of the goniometer. The closely spaced points at β = 0 correspond to a normal tomography geometry. (*c*) A 3D rendering of the reconstructed sample, showing the shape and orientation of the reconstructed symmetry axis. A zoomed-in view is shown in Fig. 6(*a*). The dashed circle marks the position of the voxel of interest plotted in Fig. 3.

The WASTT experiment consists of scanning the sample in a 2D raster grid through a focused beam to generate a stack of diffraction patterns that together make up a single projection of the sample. The raster scan is repeated for different sample orientations to create a set of projections. A sketch of the experimental geometry is given in Fig. 1 ▸(*a*). The experiments were carried out at the cSAXS beamline at the Swiss Light Source with a photon energy of 18 keV and a sample-to-detector distance of 0.2 m using a PILATUS 2M detector (Henrich *et al.*, 2009 ▸).

The X-ray beam was focused onto the sample to a spot of approximately 50 × 50 µm. The raster scans were performed using two linear stages with continuous movement in the vertical direction. Exposures were spaced 50 µm apart both vertically and horizontally. The sample was rotated using a purpose-built goniometer consisting of two orthogonal rotation stages described by Liebi *et al.* (2018 ▸). The outer rotation stage only reached angles up β = 45°, meaning that the full range of projection directions could not be measured.

An exposure time of 0.1 s was used for each diffraction pattern. The total scan time was 28 h, which includes about 6 h of overhead due to sample movement and experimental control software.

### 2.2. Sample

**Figure 2:**
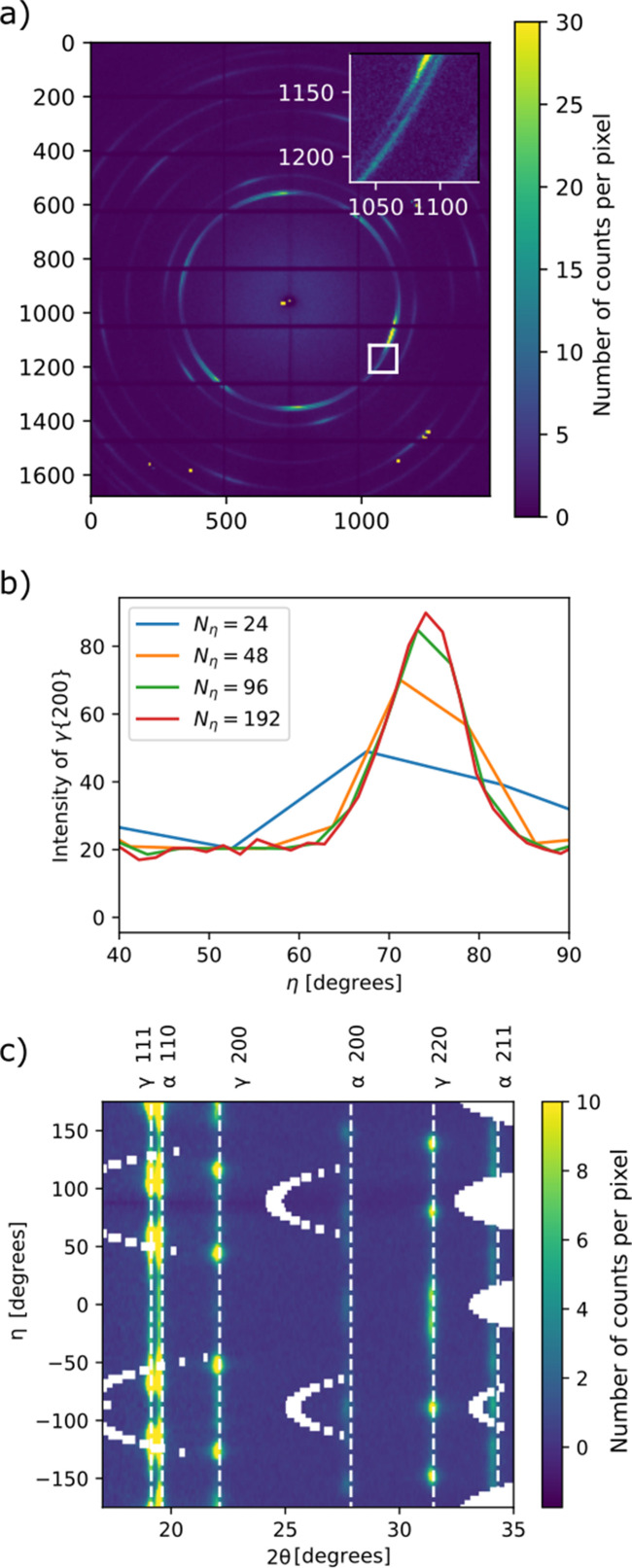
(*a*) Example detector frame with an inset showing a zoomed-in view of the region marked by a white square. The powder ring appears smooth and does not show spots corresponding to individual grains. (*b*) Integrated intensity of a section of the γ 200 peak as a function of azimuthal angle plotted with different choices of *N*_η_. (*c*) Azimuthally regrouped intensity showing the indexed peaks with *N*_η_ = 96. The colormap is oversaturated to improve visibility of the weaker peaks. The missing points in (*c*) are masked-out data points due to the gaps between the individual detector segments.

For this study we use a sample consisting of a drawn wire of ALSi 302 stainless steel of thickness 25 µm (Goodfellow Cambridge Limited, Huntington, England). The wire was prepared into a tangled knot with a total extent of approximately 3 × 3 × 3 mm to have a non-trivial spatial structure. The sample gives rise to both face- (f.c.c., γ) and body-centered cubic (b.c.c., α) diffraction peaks and has strong texture, which is seen from the fact that the individual diffraction rings have up to six separate maxima along the azimuth with peak widths on the order of 10° FWHM, as can be seen in Fig. 2 ▸.

We chose this sample because its texture is well known and can be correlated at every point to the real-space direction of the wire. Furthermore, the pole figures approximately have rotational symmetry around the wire direction, which is required by one of the applied algorithms. Finally, since the wire is thin, we can assume that any given measurement averages over the full thickness of the wire. This is the case for the shown experiments where we use a wire with 25 µm diameter and a 25 µm spot size.

In such a wire, the γ grains are known to preferably align with (111) along the wire direction. Because the probed diffraction peaks have multiplicity higher than 2, this preferred alignment will lead not just to a single direction of maximum intensity, as has been observed for the apatite 200 peak in bone (Grünewald *et al.*, 2020 ▸, 2023 ▸), but to several circles of high intensity in the pole figures centered on the wire direction. The opening angles of the circles are given by the relative angles of the corresponding reflection to the {111} directions. This provides a challenge for the existing reconstruction algorithms, as high angular resolution is needed to resolve these circles, the distances between which is on the order of 60°. The symmetry also results in the disappearance of the ℓ = 2 component of the pole figures (Bunge, 1982 ▸), which means the eigenvector-based analysis of preferred orientations (Nielsen *et al.*, 2023*b* ▸) cannot be applied here.

## 3. Reconstructions

A typical WASTT/SASTT experiment creates a large data set, typically of the order of a million diffraction patterns. Reconstructing a 3D tomogram therefore requires efficient algorithms. In this section, we present a selection of the most common reconstruction algorithms and discuss their generalization to samples with fast angular variation of the scattering patterns.

### 3.1. Description of the geometry

The sample is raster-scanned through a pencil beam while the scattered X-rays are measured on a 2D pixel detector downstream of the sample. The sample is rotated around its center by the rotation *R*_*p*_ for *p* = 0, 1, 2,…, *N*_*p*_ − 1 and the raster scan is repeated at every orientation. We describe the experiment in a sample-fixed coordinate system, (*x*, *y*, *z*), with axes as drawn in Fig. 1 ▸. When 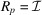, the identity rotation, the sample-fixed coordinate system coincides with the laboratory coordinates, (*x*_l_, *y*_l_, *z*_l_). *z*_l_ is the direction of the X-ray beam. A detector segment that measures a given normalized *q*-space component 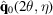 at 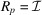 measures the *q*-space direction 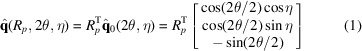
in the sample-fixed frame at a general orientation *R*_*p*_. This equation differs from SASTT only by the inclusion of the 2θ/2 factors which account for the curvature of the Ewald sphere. The scattering from a given voxel is assumed to be a function of this vector, and the measured scattering is a sum of the scattering from all voxels along the path of the direct beam times the transmission of the incoming and outgoing beams at each position and angle.

An experimental data set contains a set of raster scans at sample rotations *R*_*p*_ for *p* = 0, 1, 2,…, *N*_*p*_ − 1. Each raster scan consists of *N*_*j*_ by *N*_*k*_ points. The sample rotations used in the experiment can be parameterized by two Euler angles, α and β, corresponding to an inner (around laboratory *y* at β = 0) and an outer (around laboratory *x*) mechanical rotation stage, respectively.

The first step of analysis is to perform binning of the detector images into the polar coordinates, 2θ and η. Choosing the number of azimuthal bins, *N*_η_, is a critical step of the analysis. We perform integration in the 2θ direction over the full width of a given diffraction order, *hk*ℓ. Reconstructions are performed independently for each *hk*ℓ, so this leaves us with a data set for a given reflection of the size 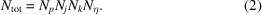


The reconstruction is a voxel map of parameter vectors where each parameter vector defines a model of the directional dependence of the scattering stemming from that voxel. The reconstructed voxel map consists of *M*_*x*_ by *M*_*y*_ by *M*_*z*_ voxels for a total of 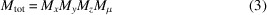
degrees of freedom. *M*_μ_ is the number of parameters describing the pole figure of a given voxel. It is an important quantity for this study as it determines how well scattering with fast angular variation can be modeled. In the next section we present a number of different reconstruction algorithms and derive an expression for how the angular resolution of the reconstruction relates to the sampling of projection directions.

### 3.2. Reconstruction algorithms

In this paper, we investigate three distinct algorithms from the SASTT literature that are all in principle capable of describing scattering from samples with arbitrarily strong texture by including sufficiently many basis functions and look at how they perform on a sample with fast directional variation in the scattering pattern. The differences between the three methods are primarily the parametrization of the functions that describe the directional dependence of scattering from each voxel. Before we go into the specifics of the three chosen methods, we first provide a broad overview of the existing algorithms in the SASTT literature.

The method described by Schaff *et al.* (2015 ▸) splits the data set into several smaller data sets corresponding to scattering along a set of discrete directions covering the unit (half) sphere and performs independent reconstructions for each such direction. In this paper, we refer to this method as discrete directions (DD).

In grating-based TT, the reconstruction algorithms rely on a model that includes the experimental broadening of the scattering due to the experimental sensitivity. The reciprocal-space model is a superposition of this function aligned along a number of sensitivity directions, of which commonly there are seven (Malecki *et al.*, 2014 ▸; Kim *et al.*, 2022 ▸). The function used to model the sensitivity is expressed as a second-order polynomial in the Euclidean coordinates and is equivalent to second-order spherical harmonics or a rank 2 tensor description.

A recent algorithm investigated by Nielsen *et al.* (2023*b* ▸) expands the local directional dependence in a sum on real spherical harmonics up to a maximum order ℓ_max_ and optimizes the full set of spherical harmonics coefficients. In the following sections, we will call this method simply spherical harmonics or SH.

The method described by Liebi *et al.* (2015 ▸, 2018 ▸) uses only the spherical harmonics with *m* = 0, called the zonal harmonics, to describe the scattering pattern. It thereby enforces that the scattering locally has axial symmetry. The direction of the symmetry axis is found as a part of the optimization, along with the spherical harmonics coefficients. We refer to this method as zonal harmonics or ZH throughout this paper.

The algorithm investigated by Gao *et al.* (2019 ▸) describes the scattering using a rank 2 tensor which is equivalent to second-order spherical harmonics, and it is therefore limited to certain smooth textures. While this method could in principle be extended to higher-order tensors, such a method would be similar to the SH method and is therefore not pursued here.

In this paper, we focus on the three models DD, SH and ZH and investigate how these models perform on samples with fast azimuthal variation in the observed scattering patterns. In these models, the directional resolution can be tuned by choosing a hyperparameter of the reconstruction. For DD, this parameter is the number of sensitivity directions as well as a selection rule that determines whether or not a given measurement point will be included in the reconstruction. For SH and ZH, the hyperparameter is the maximum order of spherical harmonics used in the expansion, ℓ_max_.

In the following subsections we go through each of the three methods in detail and investigate how the angular resolution and the number of parameters are connected, and what implications this has for the experiment and the reconstruction. We derive rules describing the sampling needed to reconstruct at a given resolution. However, as with similar sampling rules in computed tomography (Hansen *et al.*, 2021 ▸) and texture goniometry (Bunge, 1982 ▸), slight under-sampling will lead not to catastrophic failure of the reconstruction but rather to gradual degradation of the results. The rules are therefore to be understood as approximate guidelines to help when planning experiments.

### 3.3. Discrete directions

In DD the problem is split into a number of independent problems by first defining a grid of directions covering the unit half-sphere 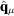 for μ = 0, 1, 2,…, *M*_μ_ − 1. Given a full data set, *I*(*j*, *k*; *R*_*p*_, η), for a certain reflection, *hk*ℓ, we seek only a subset of the data that corresponds to scattering along a certain direction in reciprocal space on the sample-fixed coordinate frame. Each such subset of the data points, *I*_μ_, constitutes a data set for an independent 3D scalar tomography problem. As such, existing algorithms from scalar 3D tomography can be used to perform the reconstruction.

For a specific reconstructed direction 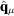, the condition that a measurement in (*R*_*p*_, η) space can be included in the given data set is that the probed direction in the sample coordinate system is close to 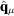, *i.e.*
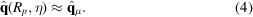

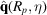
 parametrizes the circle of directions that satisfy the Bragg condition for the sample orientation *R*_*p*_. If η is treated as a continuous variable, this equation can be solved for any 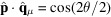, where 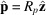 is the projection direction in the sample coordinates. This forms a circle in 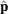 space where the angle between 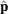 and 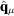 is 90°. Thus, an ideal data set where all possible projection directions can be probed constitutes a laminography data set with tilt angle θ.

In practice, only discrete points in 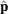 space can be sampled and equation (4) can only be approximately satisfied. To make the data sets, we need a rule to determine whether a given projection is close enough to 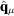 to be included or not. If we assume that the pole figure is well described by a grid of *M*_μ_ separate scattering directions uniformly distributed on the half-sphere, the average area covered by any given direction is 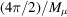 and the typical distance between two neighboring points is 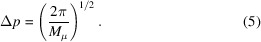


This means that, if two points in the unit sphere are closer to each other than this distance, we expect them to have similar values and we can use this as a criterion for approximately solving equation (4).

This criterion defines a band of area *A*_band_ ≈ 2π(2Δ*p*) for each 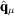, where 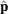 can lie. Assuming that the measured projection directions are equally spaced over the half-sphere, there will on average be 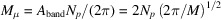 projections in this band, where *M*_μ_ denotes the number of projections in the μth data subset. To proceed, we assume that the reconstructed volume is cubic and that the raster grid is quadratic with the same number of points, *i.e.M*_*x*_ = *M*_*y*_ = *M*_*z*_ = *N*_*j*_ = *N*_*k*_. We need approximately *M*_μ_ = *N*_*j*_ to have a well constrained tomographic problem. Substituting the previous expression and rearranging yields 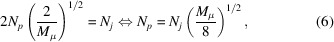
which sets a lower limit for the number of projections needed for a well constrained data set at a given resolution determined by the number of grid points *M*_μ_. Equation (5) gives an estimate of the angular resolution of the reconstruction.

For the individual *M*_μ_ scalar reconstructions, we use the SIRT algorithm as implemented in the *ASTRA Toolbox *(van Aarle *et al.*, 2015 ▸, 2016 ▸). We use the 3D GPU parallel-beam implementation (Palenstijn *et al.*, 2011 ▸) to compute the discrete X-ray transforms.

### 3.4. Spherical harmonics

A typical basis for real-valued functions on the unit sphere are the real spherical harmonics (just ‘spherical harmonics’ from here on). The spherical harmonics are enumerated by two integers, ℓ and *m*, where ℓ ∈ [0, ∞[ and *m* ∈ [−ℓ, ℓ]. A given spherical harmonic is typically denoted by the symbol 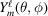 as a function of two polar coordinates. To avoid confusion regarding the symbol θ, which we use to represent the half scattering angle, we instead write the harmonics as a function of a normalized vector 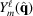.

Using the spherical harmonics as a basis set, an arbitrary square-integrable function on the sphere can be expanded as 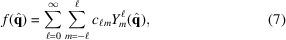
where the sum over ℓ can be truncated to a finite integer ℓ_max_ to give a finite-resolution approximation to the function. The real numbers *c*_ℓ*m*_ are called the spherical harmonics coefficients (from now just ‘coefficients’).

We will assume that the pole figures have Friedel symmetry, *i.e.*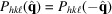, which means that all coefficients with odd ℓ are equal to zero. Counting the number of non-zero coefficients up to a maximum even order ℓ_max_ gives a total of 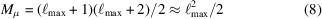
independent coefficients, where the last expression is appropriate in the limit of high ℓ_max_.

Flattening the data set into a vector, **I**, and the coefficient voxel arrays into a vector **c**_ℓ*m*_, we can write the full forward model as 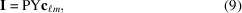
where P is a projection operator that transforms the (*x*, *y*, *z*) voxel space into (*p*, *k*, *j*) projection space. Y is a matrix containing the values of the spherical harmonics evaluated on the points probed by the experiment given equation (1). This is a linear problem, and the solution is found by minimization of the function
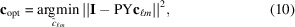
where || · ||^2^ denotes the Euclidian norm.

Let us again consider a typical experiment where the raster scan is quadratic and the reconstructed voxel grid is cubic, *i.e.* with the same number of points along all directions. We assume that the pole figure is smooth and can be represented well by a spherical harmonics expansion up to maximum order ℓ_max_. When a band-limited function on the sphere, *i.e.* a function that can be exactly represented by a spherical harmonics expansion to a given order ℓ_max_, is sampled along a circle, the resulting curve is a band-limited function in terms of linear harmonics. Therefore, we can invoke the Nyquist–Shannon sampling theorem which tells us that any *N*_η_ > 2ℓ_max_ number of measurements along a circle is sufficient to capture the full information content. Choosing critical sampling with ℓ_max_ and setting *N*_tot_ ≥ *M*_tot_ yields a lower bound to the number of projections that need to be measured to have an over-constrained problem: 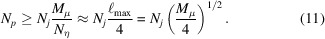


An upper limit to the angular resolution of such a reconstruction is given by the Gabor limit:
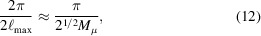
which limits the smallest features that can be represented by a band-limited function. This is the same scaling as was found for the DD method in equation (6) but with a different pre-factor.

### 3.5. Zonal harmonics

Zonal harmonics, like SH, uses a model based on spherical harmonics coefficients to represent the local pole figure but, unlike SH, ZH assumes that the local pole figure of a single voxel has axial symmetry around a direction parameterized by two angles, Θ and Φ, which are found as a part of the optimization problem. This assumption reduces the number of parameters that need to be refined in each voxel from (ℓ_max_ + 1)(ℓ_max_ + 2)/2 in SH to ℓ_max_/2 + 1 coefficients and two polar coordinates in ZH for the same ℓ_max_. The reduction in the number of degrees of freedom approaches a factor of ℓ_max_ and therefore becomes more significant at higher directional resolution.

In order to express the model in a compact form, we make use of Wigner D matrices, which are introduced in Appendix *A*[App appa]. We write the forward model as
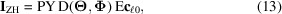
where D(**Θ**, **Φ**) is a sparse matrix containing the elements of the Wigner D matrices and **Θ** and **Φ** are vectors containing the rotation angles for all voxels of the reconstruction. E is a sparse binary matrix that expands the zonal coefficients, **c**_ℓ0_, into the full space of coefficients. This expression has the shape of an encoded version of SH, where the linear encoding step depends non-linearly on the two angle parameters. The model is slightly adapted from the original given by Liebi *et al.* (2015 ▸) to make the comparison with SH clearer and to make generalization to large ℓ_max_ easier.

As for SH, we have to choose the number of azimuthal bins of the radial integration and, as before, a number *M*_μ_ = 2ℓ_max_ is sufficient. The number of independent parameters of the model is ℓ_max_/2 + 3. Since both the number of channels and the number of parameters grow linearly with ℓ_max_, repeating the same analysis as was done for SH would suggest that a number of projections corresponding to what is needed for a scalar tomography problem would be enough to yield an over-constrained problem at an arbitrarily high ℓ_max_. As such, ZH does not suffer from the same problem as DD and SH where the number of projections needs to increase linearly with the angular resolution of the pole figures.

The man disadvantage of ZH compared with the two other methods investigated here is the non-linearity in the forward model, which means that the cost function is non-convex. This results in the optimizer often converging to local minima of the cost function that give unsatisfactory reconstructions of the sample. Previous publications describe how this challenge is overcome by utilizing prior knowledge of the sample to generate a starting guess and using heuristics, like sequentially refining the angles and the coefficients separately (Liebi *et al.*, 2018 ▸). A recent paper (Nielsen *et al.*, 2023*b* ▸) also reports on using multiple random starts and the mean of the converged solutions to yield better results. Here, we generate a starting guess from the SH reconstruction using the approach described in Appendix *A*[App appa].

### 3.6. Reconstructions

The experimental data set consists of raster scans with side lengths *N*_*j*_ = 54 and *N*_*k*_ = 62 and a total of *N*_*p*_ = 211 different orientations measured. For each reconstruction, we have to choose a number of hyperparameters of the reconstruction. This choice can be guided by the analysis presented in the previous section.

Using the inequality in equation (6) we see that DD should be able to reconstruct *M*_μ_ = 100 independent directions, giving an angular resolution of 14°. We found that increasing this number to *M*_μ_ = 300 still yields good tomograms and improves the visual quality of the pole figures. This model has a total of 65 million model parameters, around 200 000 for each of the 300 separate tomography problems. The individual tomography problems have 21 independent projections on average, giving a total of 15 million data points.

For SH, the inequality in equation (11) suggests that with the given sampling we can reconstruct up to a maximum order of ℓ_max_ = 16, which requires a number of angular bins *N*_η_ ≥ 32. To assess whether this is enough to resolve the observed diffraction patterns, we compare azimuthal curves made with a varying number of azimuthal bins [Fig. 2 ▸(*b*)]. We conclude that a number of *N*_η_ = 48 is necessary to capture most of the variation in the signal.

This choice of parameters leads to a total number of 34 million data points and 33 million model parameters. The angular resolution implied by the Gabor limit is 11°.

For ZH, we have no similar restrictions to the resolution but can, in principle, choose ℓ_max_ freely. In practice, however, ℓ_max_ and *N*_η_ are limited by the computational resources. We choose to double the numbers used in SH, ℓ_max_ = 32 and *N*_η_ = 96, leading to a total number of 78 million data points and 7.5 million model parameters. This gives a maximum angular resolution of 6°.

In summary, DD reconstructs individual diffraction directions, which makes the implementation of the reconstruction easier and more efficient and provides a simple way to implement a non-negativity constraint. SH and ZH both utilize spherical harmonics, which provide a natural way to describe smooth textures with a small number of independent coefficients. The ZH algorithm assumes that the structure of the sample has axial symmetry on the length scale of a single voxel, which limits its applicability to certain sample systems and resolutions but reduces the number of independent coefficients when applicable. Furthermore, the ZH model is non-linear, which necessitates a strategy to overcome local minima in the cost function.

## 4. Results

**Figure 3:**
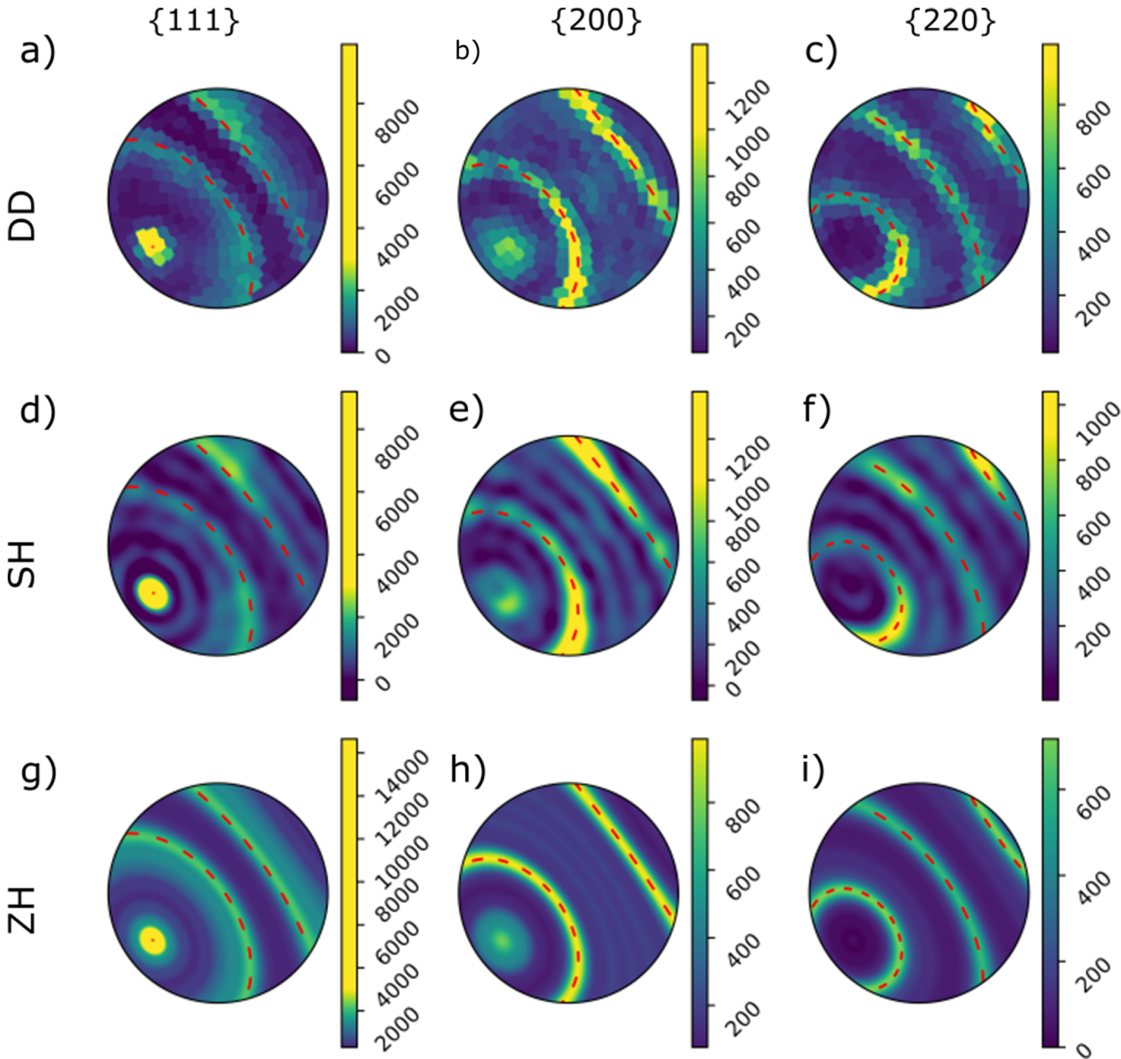
Pole figures for a single voxel reconstructed with, respectively, the (*a*–*c*) DD, (*d*–*f*) SH and (*g*–*i*) ZH algorithms. The reconstructions are of the γ phase (*a*, *d*, *g*) {111}, (*b*, *e*, *h*) {200} and (*c*, *f*, *i*) {220} pole figures. Dashed red lines show the theoretically expected positions of circles of high intensity around the fiber axis. The fiber axes in (*a*–*f*) were determined independently for each pole figure using the approach of Appendix *A*[App appa]. The colormaps of the {111} pole figures are oversaturated to make lower-intensity features visible. The position of the voxel is marked with a red circle in Fig. 1 ▸(*c*). Pole figures are plotted in stereographic projection in the sample-fixed coordinate system with the positive **x** direction pointing right, the positive **y** direction pointing up and the positive **z** direction in the center.

**Figure 4:**
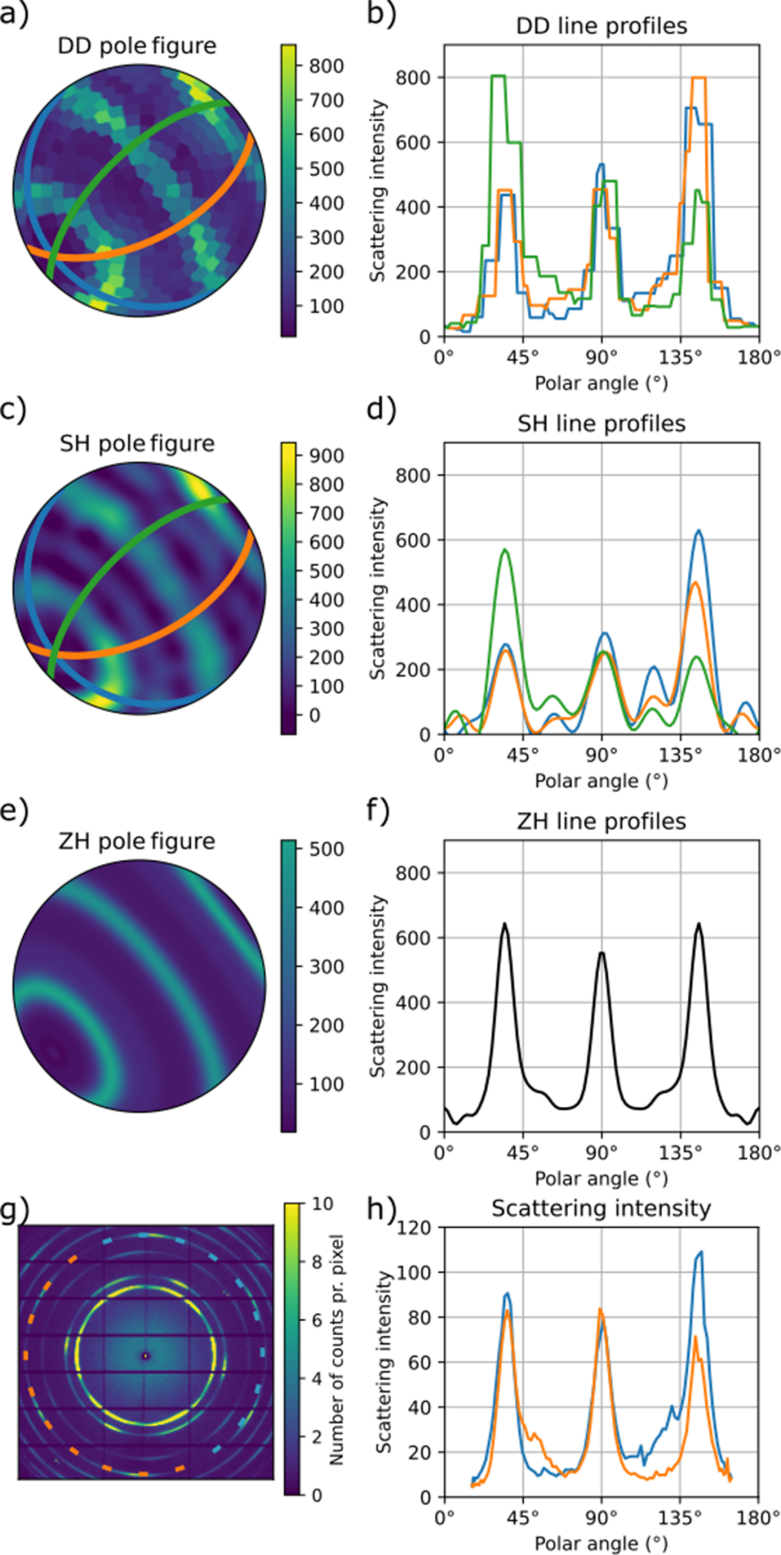
Single-pixel (*a*, *c*, *e*) pole figures and (*b*, *d*, *f*) line plots of the reconstructed value of the γ 220 peak intensity using the three different algorithms: (*a*, *b*) DD, (*c*, *d*) SH and (*e*, *f*) ZH. (*b*, *d*, *f*) show curves of intensity along the half-circle orthogonal to the fiber axis. In (*b*, *d*) three different curves are plotted, corresponding to circles 60° apart. The positions of the circles are marked in (*a*, *c*) with the corresponding colors. The ZH reconstruction is aziumthally symmetric, so here all such curves are identical and only a single curve is plotted in (*f*). (*g*) shows a single frame of the data set taken from a position where the wire direction is orthogonal to the incident beam and where the wire direction could be determined by inspecting the raster scan. (*h*) shows the intensity of the 220 peak from (*g*) as a function of the angle between the probed **q**-space component and the wire direction plotted for the left and right sides of the diffraction pattern, as marked in (*g*) with dashed lines of corresponding color.

Fig. 3 ▸ shows the calculated pole figures for a single voxel for the reconstructions using the full data set. We plot the pole figures for the three fully covered γ peaks. All reconstructions show the expected wire symmetry, and we can determine the axis of rotational symmetry with the approach described in Appendix *A*[App appa]. For the DD reconstructions, the reconstructed pole figures were first expanded into spherical harmonics before the wire direction could be determined using the same method. The pole figures contain circles of maximum intensity that show good agreement with the theoretical expectation plotted with red dashed lines. Both the DD and SH reconstructions show approximate axial symmetry, but some azimuthal variation can be seen in Fig. 4 ▸.

The pole figures of the SH reconstructions contain additional circles in the recovered pole figures aside from those expected, as can be seen in Figs. 4 ▸(*c*) and 4 ▸(*d*). We interpret these to be ringing artifacts caused by the truncation of the spherical harmonics expansion. In DD no such rings are observed, and in ZH, where the maximum order is higher, these artifacts are effectively suppressed, appearing with higher frequency and lower amplitude [Figs. 4 ▸(*e*) and 4 ▸(*f*)]. Ringing artifacts are often reported in the texture literature, and different approaches have been developed to suppress them (Bunge, 1982 ▸).

To further evaluate the reconstructed pole figures, we compare them with the measured diffraction patterns from a point in the data set where a section of the wire is orthogonal to the incident beam, identified by an approximate inversion symmetry of the diffraction pattern [Fig. 4 ▸(*g*)]. At this point, we can uniquely determine the wire axis from the 2D projection and therefore locate each azimuthal bin relative to the wire axis. We plot the azimuthal variation of the 220 peak [Fig. 4 ▸(*h*)] and compare this with the line traces of single-voxel pole figures [Figs. 4 ▸(*b*), 4 ▸(*d*) and 4 ▸(*f*)]. Doing so, we see that the features reconstructed by SH are too wide, with an FWHM of the central peak of 16.4° in contrast to 12.7° for DD, 12.7° for ZH and 9.9° for the measured diffraction. The measured diffraction pattern has 32% of the intensity in the central lobe in contrast to 29% for DD, 28% for SH and 29% for ZH. For an ideal texture where the (111) direction is fully aligned with the wire axis, half of the 220 intensity lies in the equatorial ring of the {220} pole figure, and after accounting for differences in solid angle, 37% of the intensity is expected to lie in the middle lobe. The measured data show large variability between the left- and right-hand sides of the diffraction pattern, indicating that the assumption of axially symmetric texture used by ZH is not exactly fulfilled on the length scale of the beam size. Both DD and SH reconstruct pole figures with large variations around the wire axis, similar to what is observed in the measured diffraction pattern.

**Figure 5:**
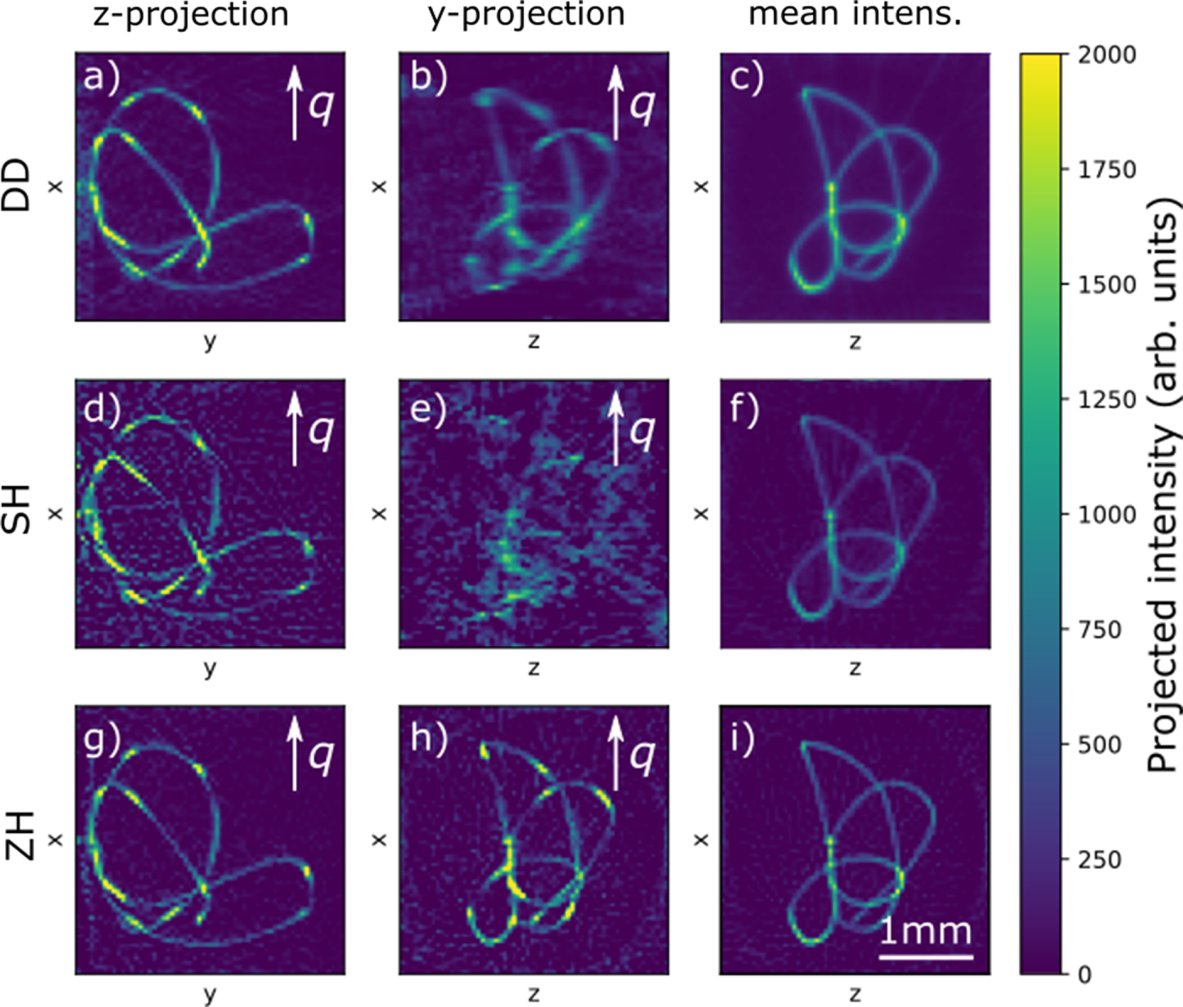
Projections of the reconstructed directional scattering density of the γ 200 peak using the three different algorithms: (*a*–*c*) using DD, (*d*–*f*) using SH and (*g*–*i*) using ZH. (*a*, *d*, *g*) show the 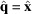 scattering directions projected along the **z** direction and (*b*, *e*, *h*) show the same scattering direction projected along the **y** direction. (*c*, *f*, *i*) show the scattering density averaged over all directions projected along the **y** direction. The projections of the directional scattering have bright regions, where the angle between the wire axis and the probed 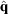 falls on the maxima of the pole figure, while the averaged density is constant along the wire.

In Fig. 5 ▸, we show three orthogonal projections of the reconstructed scattering along the **x** direction. The scattering density appears well reconstructed in the **z** projection, which is close to a projection direction that has been measured as a part of the original data set. The **y** projection could not be measured because the geometry of the goniometer blocks X-rays from this direction. This leads to the typical limited angle artifacts in Figs. 5 ▸(*b*) and 5 ▸(*e*), which is a common problem in SASTT and has previously been discussed in the literature (Schaff *et al.*, 2015 ▸; Nielsen *et al.*, 2023*b* ▸). While the missing projection angles lead to poor reconstructions of the directional scattering density, the anisotropic component is still well reconstructed, leading to the sharp images in Figs. 5 ▸(*c*) and 5 ▸(*f*).

In the ZH model, scattering in different directions cannot vary independently, because rotational symmetry has to be maintained. This effectively leads to a sharper image in Fig. 5 ▸(*h*) compared with the corresponding image made with the SH [Fig. 5 ▸(*e*)] and DD [Fig. 5 ▸(*b*)] algorithms.

Comparing the images made with the DD [Figs. 5 ▸(*a*)–5 ▸(*c*)] and SH [Figs. 5 ▸(*d*)–5 ▸(*f*)] algorithms, we see that the SH images contain a large amount of noise seen as intensity outside of the silhouette of the wire sample. We interpret this as coming from overfitting of the high-ℓ components of the reconstruction. In DD, these overfitting features have to some extent been avoided by implementing a non-negativity constraint on the reconstruction. This is an advantage of the DD method over SH, as a non-negativity constraint cannot easily be formulated for spherical harmonics expansions (Nielsen *et al.*, 2023*b* ▸). The non-negativity constraint is more useful when many voxels have zero scattering and the solution therefore lies on the constraint boundary. The benefit offered by the non-negativity constraint is therefore expected to be less drastic for more dense samples.

**Figure 6:**
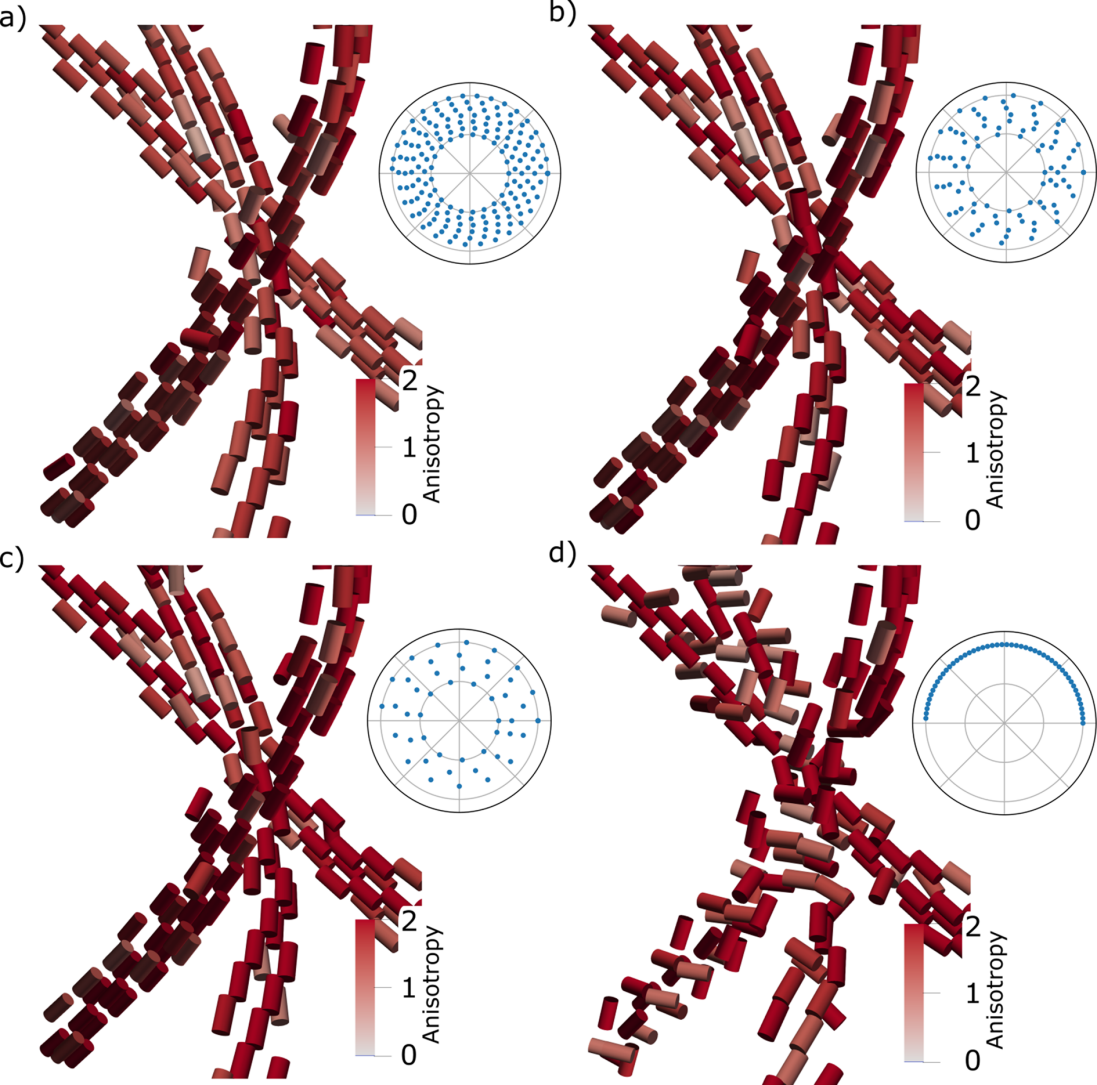
Comparison of reconstructions with the ZH method and using SH to provide a starting guess for the optimization using different subsets of the full data set containing (*a*) 178 projections, (*b*) 89 projections, (*c*) 49 projections and (*d*) 51 projections. In (*a*–*c*), the projections are selected to make the sampling of the unit sphere as evenly spaced as possible, while in (*d*) only a single equatorial line is used. The plotted voxels are selected with a volume mask based on a threshold of the absorption tomogram. The 3D renderings use perspective shading with the virtual light source placed above the scene. The anisotropy used as the color scale is the anisotropic power divided by the isotropic power 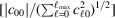.

The ZH model has far fewer degrees of freedom than the SH models. We therefore see that the overfitting artifacts are less apparent in Figs. 5 ▸(*g*)–5 ▸(*i*) compared with Figs. 5 ▸(*d*)–5 ▸(*f*). The reduced number of degrees of freedom also means that we expect to be able to achieve good reconstructions with a smaller number of measurements, as was shown in the previous section. However, because the ZH model is non-convex, we need a good starting point for the angle parameters to achieve convergence to the global minimum. In Figs. 6 ▸(*a*)–(*c*) we compare the reconstructions achieved by first creating a starting guess using SH and then performing the ZH reconstruction. We use a progressively smaller subset of the full experimental data set in both the SH and ZH algorithms. Even with the smallest number of projections (49), we are able to recover the wire orientation. We also perform a reconstruction using only the measurements from a normal tomography geometry, with β = 0. In this reconstruction, we see that the reconstructed symmetry axis does not align with the wire axis, supporting the assertion that tensor tomography benefits from projection directions spaced over the whole unit sphere and not just a single equatorial band (Liebi *et al.*, 2018 ▸).

## 5. Discussion

Both DD and SH succeed in reconstructing pole figures displaying the expected circles of high intensity and in re­covering a symmetry axis that aligns with the wire direction. While the two methods obtain reconstructions of similar quality, the DD approach has some practical advantages in that it splits the TT problem into a number of much smaller scalar tomography problems that can be solved more efficiently with smaller demands on the computational resources needed. Furthermore, because the algorithms and software for scalar CT are more established, many reconstruction algorithms and software packages are available that can readily be used to perform the reconstructions, whereas new algorithms for TT have to be developed, implemented and tested from scratch. Yet shortcomings of DD are clearly visible when considering scattering in directions where a full circle of projections cannot be collected [Figs. 5 ▸(*b*) and 5 ▸(*c*)]. And while the implementation of SH used here is kept basic, methods such as SH that perform a global optimization over all scattering directions could be adapted to utilize correlations of the scattering in different directions, which we expect would alleviate this problem.

In ZH, scattering in different directions is not reconstructed independently, because the sample is assumed to be rotationally symmetric locally. This prior knowledge of the sample symmetry gives a huge reduction in the number of independent parameters, which is probably responsible for the better performance of the ZH reconstruction compared with the two other investigated methods. However, for many samples of interest, such axial symmetry will not be present, and the ZH algorithms can therefore not be used as a general reconstruction tool.

While none of the algorithms tested here explicitly makes use of the fact that the sample is sparse (*i.e.* that it contains many voxels with zero scattering), the DD reconstructions use a positivity constraint, which suppresses streaking artifacts in the empty part of the reconstructed volume and helps explain the relatively good quality of the DD reconstructions compared with SH observed in Fig. 5 ▸. A significant disadvantage of the spherical harmonics basis used in SH and ZH is that there is no easy formulation of a positivity constraint, which prevents a similar constraint being applied for SH. In the original paper by Liebi *et al.* (2015 ▸), non-negativity was enforced by using a basis consisting of the square of the spherical harmonics. This approach is not pursued in the present paper because the squares of the spherical harmonics are not a complete set and can therefore only be used to model special textures.

The sample used for this study was deliberately chosen for its low absorption. TT relies on being able to express the scattered intensity as a line integral of the scattering density of the sample. This is not exactly the case when the sample absorbs, and the absorption of both the incident and scattered light has to be taken into consideration. In SASTT, this is corrected for by utilizing a complimentary measurement of the transmitted beam intensity, from which the transmission coefficient can be calculated. Because SASTT uses X-rays scattered to small angles, the absorption seen in all the scattering directions is approximately the same and equal to the absorption seen by the transmitted beam.

For WASTT, this approximation becomes less appropriate as X-rays scattered to different directions and scattered in different parts of the sample all experience different effective transmission. Computational transmission corrections can be formulated, as is discussed by Grünewald *et al.* (2023 ▸), but in general this is a difficult problem as the correction will either need to be approximate or involve the calculation of a very large number of line integrals through the sample absorption density, which increases the computational cost. This problem is alleviated by using harder X-rays, both because the absorption coefficient is smaller and because the scattering angle, and therefore the difference between the path of the transmitted and scattered beams, is smaller. In the current study, the photon energy was limited by the beamline at which it was performed, but in general WASTT would benefit from utilizing harder X-rays, as long as the diffraction rings can be measured with sufficient resolution.

We have shown here that ZH achieves satisfactory reconstructions even with a very small number of measured projections, a conclusion also reached by Liebi *et al.* (2018 ▸) for a different type of sample. This might be a result of the particularly simple sample used for this study, which has a large fraction of zero-intensity voxels. The main difficulty with applying ZH in practice is the issue of non-convexity. For the reconstructions shown here, we depend on other methods to provide a reasonable starting guess for the orientation of the symmetry axis. In the SASTT literature, the same problem is overcome by using more advanced approaches to the optimization and regularization to enforce a smooth variation of the direction (Liebi *et al.*, 2018 ▸).

The pole figures of different crystal reflections from the same crystalline phase are not independent, as they all arise from the same crystals. The different pole figures are bound to be connected to the distribution of crystal-grain orientations present in the sampled volume, which can be quantified by the orientation distribution function (ODF). The ODF in turn must respect the symmetry of the crystal lattice, which provides extra symmetry information that can, in principle, be enforced on the reconstruction. Texture analysis, such as described by Bunge (1982 ▸), provides a formalism to calculate pole figures from the ODF and *vice versa* when enough independent directions are reconstructed to make the problem well constrained.

In texture analysis, the ODF is expanded into a sum of generalized spherical harmonics. The problem of calculating the ODF in general has many more degrees of freedom than a pole figure at the same ℓ_max_ and is therefore only well constrained when many diffraction rings of the sample crystalline phase are measured. In practice, however, symmetry can decrease the number of expansion coefficients dramatically. Both sample symmetries (such as the wire symmetry) and crystal symmetries (such as the cubic symmetry of the f.c.c. or b.c.c. lattice) can be utilized in this way, whereas only the sample symmetries can be straightforwardly utilized in the expansion of the individual pole figures.

**Figure 7:**
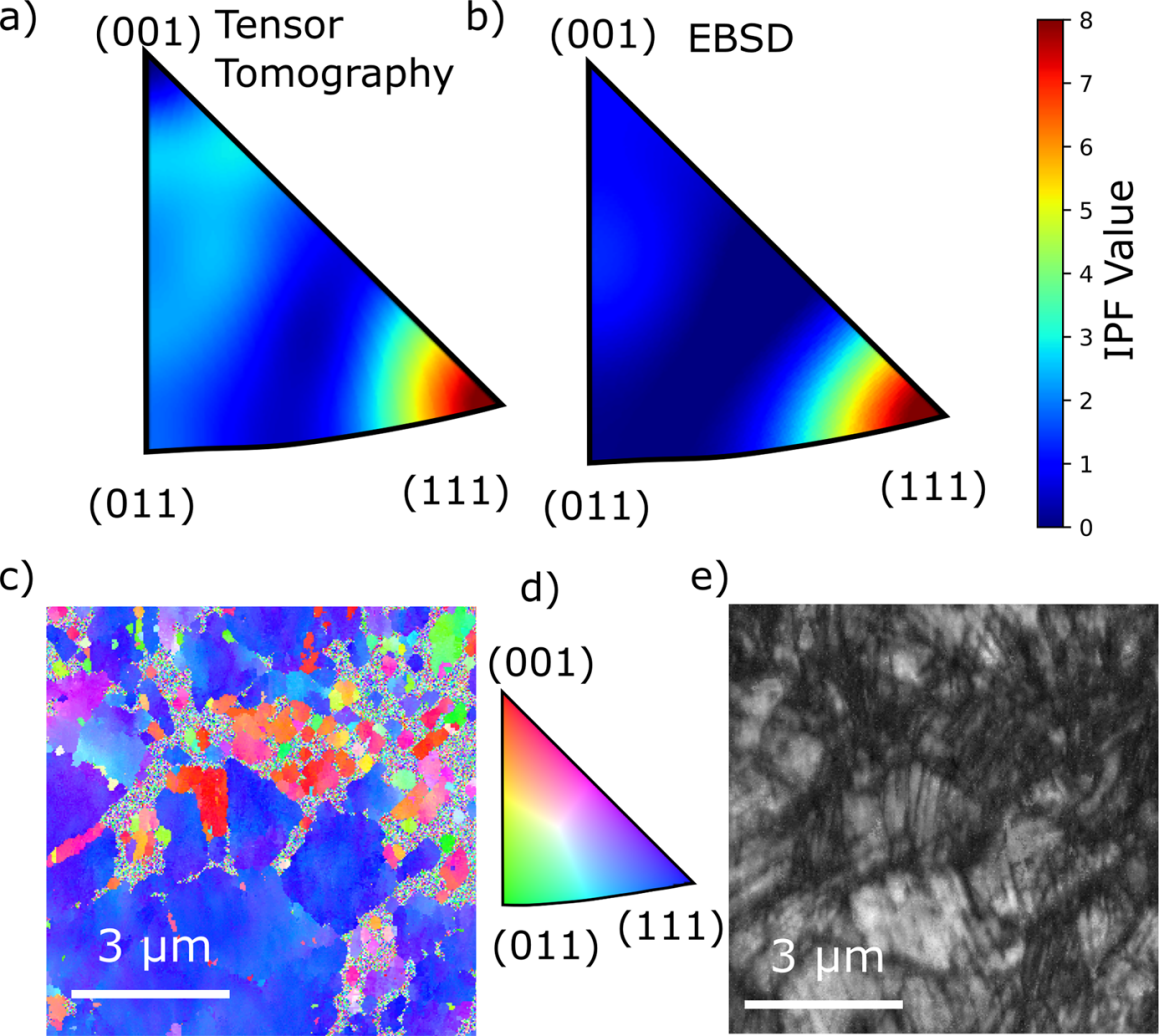
(*a*) Calculated inverse pole figure in the fiber direction. The inverse pole figure is fitted to the average coefficients of the three reconstructions made using the ZH method. (*b*) Inverse pole figure from an EBSD measurement. (*c*) Inverse pole figure map from same sample showcasing small grains sized 3 µm and below. (*d*) Legend explaining the meaning of the colors in (*c*). (*e*) Image quality map showcasing the presence of noticeable deformation within the grains.

In the special case of axial sample symmetry, the full texture information is contained in the inverse pole figure (IPF) in the symmetry direction because the ODF is independent of the Euler angle corresponding to rotations about the wire direction. Utilizing the cubic symmetry of the crystal lattice, the three reconstructed {*hk*ℓ} orders are enough to calculate this IPF up to the highest resolved order ℓ = 32, which is plotted in Fig. 7 ▸(*a*). The IPF can be seen as the probability density of finding a given lattice vector aligned along the sample direction, and, as expected for γ-steel, we see preferential alignment of the {111} direction along the wire direction. The ODF and IPF were calculated via the approach described by Bunge (1982 ▸) using the real spherical harmonics as defined by Schaeben & van den Boogaart (2003 ▸). We also performed electron backscatter diffraction (EBSD) measurements on a piece of wire from the same spool. From these measurements we calculate an inverse pole figure in the wire direction, which is shown in Fig. 7 ▸(*b*). Like the IPF calculated from the tomographic reconstruction [Fig. 7 ▸(*a*)], it also displays a maximum at (111) with approximately the same angular spread. The EBSD mapping was done on a surface of 9 × 15 µm normal to the wire direction and it therefore averages over far fewer grains compared with the 50 µm cubed volume of a reconstructed voxel. The IPF map is presented in Fig. 7 ▸(*c*) and showcases a prevalence of {111}-oriented grains. Fig. 7 ▸(*e*) shows the image quality of the collected IPF map. This map contains many areas with low image quality, which can be an indication of high deformation levels (Schwartz *et al.*, 2008 ▸).

Tensor tomography is already being applied to study a number of sample systems that display anisotropic scattering properties such as deformed metals and bio-minerals. While we have here tested the methods on wide-angle diffraction data, there are also systems that display fast angular variation of the small-angle scattering such as crystalline polymers and highly aligned fibers in composites and biomaterials.

## 6. Availability

The data used to create the reconstructions can be downloaded from https://doi.org/10.5281/zenodo.10889439, and the algorithms presented have been made available in the software package *mumott*, which is distributed under an open-source license and can be downloaded from https://mumott.org (Nielsen *et al.*, 2023*a* ▸).

## 7. Conclusion

We have shown that the TT approach can be used to reconstruct pole figures of small-grained polycrystals with strong texture. We compared a number of the most common algorithms from TT and highlighted various advantages and shortcomings of the different approaches. The results of this pilot study indicate that WASTT shows promise as a new non-destructive technique to study microstructure in small-grained polycrystalline materials, complementing existing methods for large-grained materials.

Both DD and SH give good reconstructions of the sample shape and recreate the expected features in the pole figures. They both, however, contain severe artifacts, interpreted as limited angle artifacts, due to a missing range of projection directions that could not be measured. In the DD reconstruction these artifacts are somewhat suppressed owing to the non-negativity constraint. The ZH reconstruction reduces the artifacts even further by enforcing axial symmetry on the pole figures of individual voxels. The disadvantage, however, is higher computational costs and a more complicated optimization procedure owing to the existence of many local minima in the cost function.

## Supplementary Material

X-ray scattering tensor-tomography dataset for a steel wire using the austenitic {220}-peak: https://doi.org/10.5281/zenodo.10889439

## Appendix A Wigner’s D matrices

The existing implementation of the ZH algorithm used for a number of publications (Liebi *et al.*, 2015 ▸, 2018 ▸; Grünewald *et al.*, 2020 ▸; Mürer *et al.*, 2021 ▸) is only implemented up to ℓ = 6, which is not sufficient for this paper. In attempting to extend this algorithm, we found that a great simplification of the implementation could be achieved by utilizing the approach of Wigner’s D matrices to carry out the rotations rather that the explicit rotations of polar coordinates used in the existing implementation.

In this appendix we go over the details of the new implementation, which is publicly available as part of the *mumott* software package (Nielsen *et al.*, 2023*a* ▸).

### A1. Definition of the D matrices

For a function  where  and a rotation *R* ∈ SO(3), we define the rotated function as .

One of the useful features of spherical harmonics is that the function space spanned by the spherical harmonics of a given order ℓ is closed with respect to rotation. In other words, the rotated function of any spherical harmonic with order ℓ will be equal to a linear combination of spherical harmonics only with order ℓ. That is to say some set of matrix elements, , exist such that

where on the right-hand side we treat *m* as matrix and vector indices for the matrix  and vector , respectively. This also means that the coefficients of a rotated function can be calculated from the coefficients of the original function as a matrix product:

where *c*_ℓ*m*_ are the coefficients of a function  and  are the coefficients of the rotated function.

The matrices defined this way are called Wigner D matrices. There is one (2ℓ + 1) × (2ℓ + 1) matrix for each order ℓ, and the matrix elements are continuous functions of the rotation. The Wigner matrices of combined rotations are the matrix products of the matrices of individual rotations:

which means the D matrices for a given order ℓ constitute a representation of the rotation group with a one-to-one mapping and all the same algebraic rules that are known for the 3-by-3 rotation matrices. One of these rules is that any rotation may be described through its Euler angles from a set of particularly simple rotations (the ‘primary’ rotations about the axes of the original Cartesian coordinate system *x*, *y*, *z*):

where (Φ, Θ, Ψ) constitute a set of *zyz* Euler angles. The representation property of the D matrices therefore means that the corresponding D matrix can be calculated by a similar matrix product, if the matrices of these primary rotations are known.

The elements of the D matrices are real and differentiable functions of the rotation, , and therefore also continuous differentiable functions of the Euler angles. These functions constitute a complete orthornormal set of functions on SO(3) and are commonly called ‘generalized spherical harmonics’ (Schaeben & van den Boogaart, 2003 ▸).

### A2. Calculation of D matrices

If we want to calculate the D matrices using equation (17), we only need to calculate the D matrices corresponding to rotations about the *z* and *y* axes. Rotations about the *z* axis (which we chose as the special axis in our definition of the polar coordinates) are especially easy to work with, as they only change the azimuthal coordinate and not the polar coordinate. This has the effect that rotations only mix spherical harmonics with equal |*m*|. That is to say, the Wigner D matrices become sparse and the only non-zero elements are

and

*e.g*.

A rotation about the *y* axis can be achieved by first rotating the *y* axis into the *z* axis, performing a rotation about the new *z* axis and then performing the inverse of the first rotation. We can write this as

Utilizing this approach, we only need to compute the D matrices corresponding to the rotation *R*_*x*_(π/2). While this is not easy for an arbitrarily large ℓ, there is an extensive literature on the subject and software packages that can be utilized for this purpose. The matrices  can then be precomputed and stored.

Now we can write the D matrix for an arbitrary orientation as

where (…)^T^ refers to the matrix transpose.

### A3. Derivatives

The most obvious derivatives to consider when dealing with spherical harmonics are perhaps the derivatives with respect to the polar coordinates, but for our purpose it is sufficient and maybe more convenient to consider derivatives of a rotated function with respect to the Euler angles of the rotation. For example, consider the quantity

which is itself a function on the unit sphere.

Using the D matrices, the coefficients of this derivative can be efficiently calculated by only defining the extra matrix: δD^ℓ^(*R*_*z*_(α)), with the elements

which can easily be evaluated by differentiating the sine and cosine functions of the *z*-axis D matrices. Using an identity for the differentiation of matrix products, we can now calculate the coefficients of the differentiated function using the equation

where  are the coefficients of the original function and *c*_ℓ*m*_ are the coefficients of the rotated function. This expression is the same as the normal expression for the rotation (21), just with one of the matrices replaced with the differentiated matrix. Similar expressions for the Φ and Ψ derivatives can be derived in the same way.

### A4. Reformulating ZH with D matrices

We describe the scattering by a sample with local axial symmetry with a voxel model, where every voxel contains a number of spherical harmonics coefficients  with *m* = 0, and a pair of Euler angles, Θ_0_ and Φ_0_, describing the local fiber axis. The original function has axial symmetry about the *z* axis, and the rotated function has symmetry about the axis with polar coordinates (θ, ϕ) = (Θ_0_, Φ_0_). The forward model of the ZH TT algorithms is described in a few steps:

First the *m* = 0 coefficients are expanded into a basis of all the coefficients:

where E is a binary matrix (*i.e.* a matrix containing only 0s and 1s) with only a single 1 in each line. The transpose of E is a projection matrix which projects the full spherical harmonic coefficient space onto the sub-space with rotational symmetry about the *z* axis.

Then the coefficients are rotated from the local coordinate system of the voxel, where the function is rotationally symmetric, into the sample coordinate system:

where D(*R*) is a block diagonal matrix containing the D matrices for every odd order ℓ. The voxel maps of these coefficients are integrated along the direction of the X-ray beam, here represented by a linear operator P, with a conjugate represented by P^T^. These projected coefficients are finally mapped to the detector segments by multiplying with a matrix containing values of the spherical harmonic functions evaluated at the point of the corresponding detector segments. We collect these function values in the matrix Y:

We use the least squares error

Now that the full model is stated in linear algebra terms, we can differentiate it using equations from matrix calculus:

and

Again, the Φ_0_ derivatives follow in a similar fashion.

### A5. Finding the direction of rotational symmetry

We introduce the heuristic measure for the degree of symmetry, *d*, around the *z* axis:

which is equal to the power of the rotationally averaged function divided by the power of the original function. If the coefficients of the function *f* are given, this quantity is equal to

The degree of symmetry with respect to rotation around any other direction, with polar coordinates (θ, ϕ), can be calculated using Wigner’s D matrices as

which is equal to the power of the zonal harmonic modes along the given direction divided by the total power. The direction of orientation can now be found by optimizing this quantity with respect to the two direction angles:

The optimization is performed by first evaluating *d*(θ, ϕ) on a mesh grid sized 2ℓ_max_ × 2ℓ_max_ and choosing the point with the highest value. This point is then used as a starting guess for a gradient optimization method to refine the optimal direction. The initial grid search was found to be necessary to avoid falling into a local minimum.
